# Multi-omic approach to decipher the impact of skincare products with pre/postbiotics on skin microbiome and metabolome

**DOI:** 10.3389/fmed.2023.1165980

**Published:** 2023-07-18

**Authors:** Min Li, Junhong Mao, Isabel Diaz, Evguenia Kopylova, Alexey V. Melnik, Alexander A. Aksenov, Craig D. Tipton, Nadia Soliman, Andrea M. Morgan, Thomas Boyd

**Affiliations:** ^1^Colgate−Palmolive Company, Piscataway, NJ, United States; ^2^Clarity Genomics Inc., San Diego, CA, United States; ^3^Arome Science Inc., Farmington, CT, United States; ^4^RTL Genomics, MicroGenDX, Lubbock, TX, United States

**Keywords:** skin microbiome, prebiotics, postbiotics, multi-omics, metagenomics, metabolomics

## Abstract

**Introduction:**

Although pre/pro/postbiotics have become more prevalent in dermatologic and cosmetic fields, the mode of action when topically applied is largely unknown. A multi-omic approach was applied to decipher the impact of the skincare products with pre/postbiotics on skin microbiome and metabolome.

**Methods:**

Subjects with dry skin applied a body wash and body lotion with or without pre/postbiotics for 6 weeks. Skin hydration was measured at baseline, 3 and 6 weeks. Skin swabs were collected for 16S rRNA gene sequencing, metagenomics and metabolomics analysis.

**Results:**

Skin hydration significantly increased in both groups. The prebiotic group significantly reduced opportunistic pathogens, e.g., *Pseudomonas stutzeri* and *Sphingomonas anadarae*, and increased the commensals, e.g., *Staphylococcus equorum*, *Streptococcus mitis, Halomonas desiderata*. Bacterial sugar degradation pathways were enriched in the prebiotic group, while fatty acid biosynthesis pathways were reduced in control. The changes on skin metabolome profiles by the products were more prominent. The prebiotic group performed greater modulation on many clinically-relevant metabolites compared to control. Correlation analysis showed *H. desiderata* and *S. mitis* positively correlated with skin hydration, *P. stutzeri* and *S. anadarae* negatively correlated with the metabolites that are positively associated with skin hydration improvement.

**Conclusion:**

This holistic study supported a hypothesis that the pre/postbiotics increased skin hydration through the modulation of skin microbiome, metabolic pathways and metabolome.

## Introduction

The skin is the largest organ of the human body and is colonized by a variety of living microorganisms. The microbes form an invisible ecosystem that protects the skin from external aggressors, contributes to the production of essential nutrients and educates the immune system to ensure human health (1, 2). A disruption in the microbiome can result in many skin disorders, such as atopic dermatitis, acne and psoriasis (3–5). Therefore, developing new solutions that support the structure and functionality of the skin microbiota is an emerging opportunity to manage skin health in the field of dermatology.

Pre, pro and postbiotics are becoming more prevalent for skin health through modulation of skin microbiome. Studies have shown the beneficial effect of postbiotics, which are the byproducts of probiotics, and prebiotics on skin health (6–9). Previously, we have developed a triple-biotic complex, a combination of a prebiotic (inulin), a “smart biotic” (butyloctanol) and postbiotics (lactic acid and pyruvic acid), and demonstrated the benefits of triple-biotics by inhibiting the growth of undesirable bacteria, promoting beneficial bacteria, and enhancing skin barrier *in vitro* (10). However, the mechanism of action (MOA) of such topically applied ingredients *in vivo* is not fully understood.

Understanding the impact of ingredients or products on skin microbiome requires high-throughput, holistic and high resolution techniques. Next generation sequencing has revolutionized our understanding of the microbiome diversity in our body. 16S rRNA gene sequencing builds an important knowledge base regarding the microbiome in health and disease, and remains a valuable tool for microbial community profiling (11). Technical and analytical breakthroughs in sequencing and bioinformatics have enabled shotgun metagenomics to be a cost-effective tool to explore strain diversity and the functional potential of the microbiome (12). It has dominated in gut microbiome analysis, and now expanded to skin microbiome research (13, 14). More recently, mass spectrometry-based metabolomics has emerged as a new tool to characterize the chemical makeup of the skin surface and correlate it with the microbes (15). Metabolomics focuses on exploring distributions of “small” molecules, typically <2000 Da, which are nutrients for shaping the microbial community, important byproducts of host–microbe interaction regulating host metabolic homeostasis (16) or agents of microbial “warfare,” such as microbially-produced antibiotics (17). Accessibility to multi-omics technologies has allowed for integrated analysis of 16S rRNA gene sequencing, metagenomics and metabolomics to systematically characterize the composition, function and metabolic dynamics of microbiome in relation to human diseases (18, 19). However, this type of holistic approach has been largely limited to gut microbiome research (20).

In this report, we conducted a clinical study to investigate the impact of the skincare products with triple-biotics on skin microbiome compared to a control, and used a multi-omic approach integrating 16S rRNA gene sequencing, shotgun metagenomics and untargeted mass spectrometry-based metabolomics to explore the MOA of pre/postbiotics on skin health through modulation of skin microbiome and metabolome.

## Materials and methods

### Study design and sample collection

**Figure 1:**
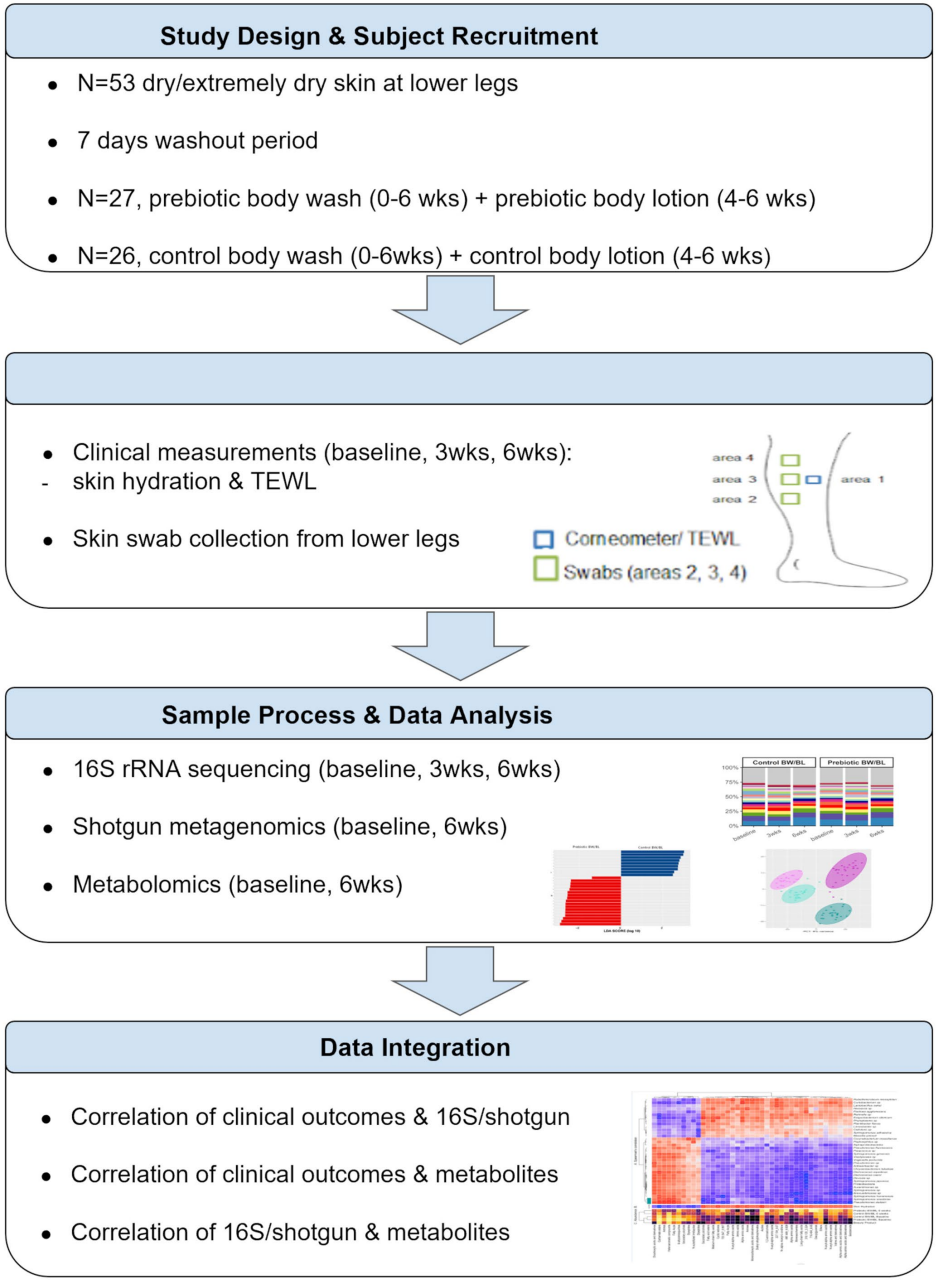
The overview of study design and data analysis workflow. TEWL, transepidermal water loss.

A randomized clinical study was conducted by ProDERM (Schenefeld, Germany) to assess the impact of the skincare products on the skin microbiome and metabolome of normal and dry to extremely dry skin. The study was approved by an independent Institutional Review Board. All the subjects signed informed consent forms. The complete study design is described in Supplementary material and Supplementary methods. Multi-omics analysis was only applied on the subjects with dry/extremely dry skin; therefore, we only presented the data from the dry/extremely dry skin group in this report. The overview of study design and data analysis workflow was illustrated in Figure 1.

Fifty-three female subjects (18–70 year-old) with dry/extremely dry skin on the lower legs were assigned to use a standard marketed shower gel (Palmolive®, Colgate-Palmolive, Piscataway, New Jersey) once daily on their body, except for their legs for 7 days during the washout period. One group (*N* = 27) was given a shower gel and body lotion with triple-biotics (Sanex®, Colgate-Palmolive). The other group (*N* = 26) continued to use the washout shower gel and a body lotion (E45 Daily Lotion, Reckitt-Benckiser®, Slough, United Kingdom). The subjects washed with the shower gel once daily in weeks 1–6, and applied the body lotion twice daily in weeks 4–6 on their lower legs. During the study, the subjects were not allowed to use any other products on their lower legs.

At baseline, 3 and 6 weeks, skin hydration was measured by Corneometer CM 825 (Courage and Khazaka, Cologne, Germany), and TEWL was measured by Tewameter® TM 300 (Courage and Khazaka, Cologne, Germany) from the lower legs. One skin swab was taken at each assessment time for 16S rRNA gene sequencing. Two swabs were collected at baseline and 6 weeks for shotgun metagenomic sequencing and metabolomics analysis, respectively.

The differences of skin hydration and TEWL between the groups were analyzed via a paired *t*-test using SAS statistical software (North Carolina). Statistical significance was defined as value of *p* < 0.05.

### 16S rRNA gene sequencing and data analysis

V1-3 region of 16S rRNA gene sequencing was conducted by RTL Genomics (Lubbock, Texas) (21). Paired reads were assembled, quality filtered, and clustered into 97% sequence similarity OTUs. Taxonomic classification was performed using an RTL Genomics classifier and in-house reference database. Product group and time point were evaluated for their relative importance on alpha and beta diversity using ANOVA and PERMANOVA, respectively. ANCOM-BC (22) procedure was used to screen differentially abundant taxa between groups. Expanded methods are provided in Supplementary material and Supplementary methods.

### Shotgun metagenomic sequencing and data analysis

Shotgun metagenomic sequencing was conducted by CosmosID (Germantown, Maryland). DNA extraction, sequencing procedure, taxonomic and functional classification analysis were performed as described in Supplementary material and Supplementary methods.

LEFSe was used to identify differentially abundant MetaCyc pathways between the baseline and 6 weeks in each product group and between the groups at 6 weeks (23). LEfSe was calculated with a Kruskal–Wallis alpha value of 0.05, a Wilcoxon alpha value of 0.05, and a logarithmic LDA score threshold of 1.0.

### Mass spectrometry-based metabolomics and data analysis

Untargeted metabolomics data acquisition using liquid chromatography mass spectrometry (LC–MS) was conducted by Arome Science, Inc. (Farmington, Connecticut) and integrated microbiome-metabolome data analysis was conducted by Clarity Genomics, Inc. (San Diego, California). The detailed description of analysis protocols is given in Supplementary material and Supplementary methods.

Clinical samples and individual skincare products were subject to untargeted mass-spectrometry data profiling. Feature detection was performed using MZmine2 (24). Feature annotation (levels of annotation summarized in (25)) using spectral matching was performed against GNPS Release 30 (26) public libraries and NIST 2020 commercial library (level 2). *In silico* feature annotation (level 3) using MS/MS data was further performed using SIRIUS v.5.0.1 (27–30) for discriminative features (web services were provided by Bright Giant GmbH; Jena, Germany). Metabolite features were removed if none of the biological sample peak abundances were higher than 3 times the maximum value in the blank samples, resulting in 63% of features retained for downstream analysis. PCA and PLS-DA analyses were performed using the R package ropls v1.24.0 (31). Permutation testing was conducted for PLS-DA models to avoid overfitting using 100 random permutations and 5-fold cross-validation. Variable Importance in Project (VIP) and the Wilcoxon Rank Sum test was used for discriminant feature selection separating groups by time and treatment, adjusted for multiple testing using the Benjamini and Hochberg correction (referred to as q-value). Putative origin of discriminant metabolites from the skincare products was examined by the Wilcoxon Rank Sum test by comparing the peak abundance between 6 weeks samples (control and product) and the skincare products. Microbiome-metabolome correlations were computed using Spearman’s correlation and *p*-values FDR-adjusted.

## Results

### Clinical outcomes

All the test groups demonstrated very good skin tolerability as per the study dermatologist evaluation during the course of the study with no product-related adverse events. Focusing on the two dry/extremely dry skin groups, after 3 weeks of body wash use only, corneometer and transepidermal water loss (TEWL) results demonstrated a significant increase in skin hydration (*p* < 0.05) along with maintenance of the skin barrier (*p* > 0.05). At 6 weeks, skin hydration was even further improved (*p* < 0.001) while maintaining the integrity of the skin barrier (*p* > 0.05). Skin hydration and TEWL are not different between prebiotic and control groups. The skin hydration measurement was shown in Supplementary Figure S1.

### Microbial composition by 16S rRNA gene sequencing

**Figure 2:**
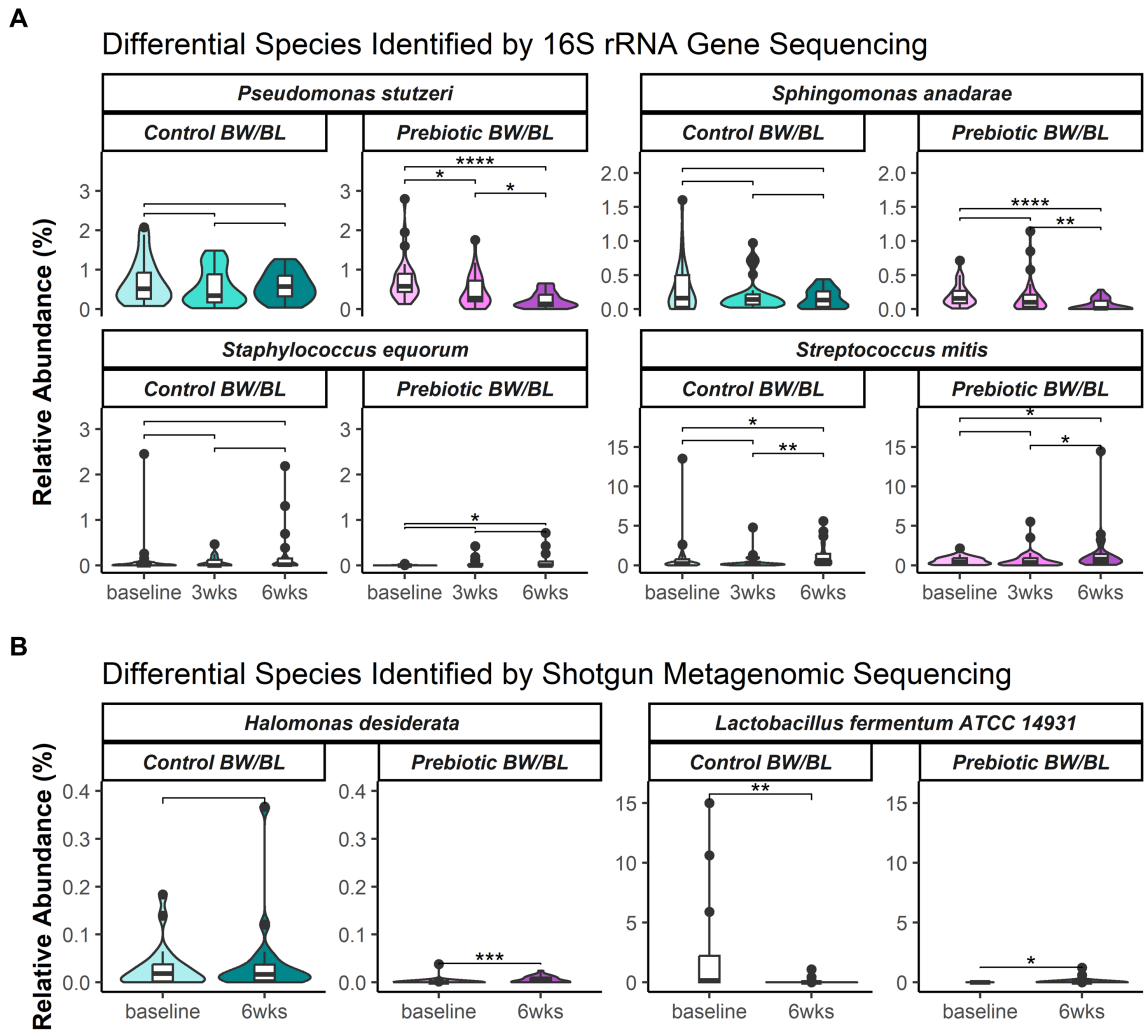
Relative abundance of select bacterial species or strains identified as significantly differentially abundant between time points and product groups, identified by **(A)** 16S rRNA gene sequencing and **(B)** shotgun metagenomics. Select species shown here and in Supplementary Tables S1, S2 were significantly differentiated per ANCOM-BC. Results of *post hoc* pairwise testing by Wilcoxon rank sum test provided to show differences per each time and within treatment groups. Significance represented as follows: ‘*’ = 0.01 < *p* < 0.05; ‘**’ = 0.001 < *p* < 0.01; ‘***’ = 0.0001 < *p* < 0.001; and ‘****’ = *p* < 0.0001. BW, body wash; BL, body lotion.

The overall microbial profiles at genus level at each time point and treatment group and for each individual were shown in Supplementary Figure S2. Alpha diversity of 16S rRNA gene sequencing data showed no significant difference between the groups in the number of observed operational taxonomic units (OTUs) and Shannon diversity index (Supplementary Figures S3A,B). Beta diversity was assessed using weighted UniFrac phylogenetic distance to summarize the microbial composition between the groups (Supplementary Figure S3C). Following global significance testing using adonis, both time (*p* = 0.001, *R*^2^ = 0.04) and product (*p* = 0.029, *R*^2^ = 0.01) were found to significantly associate to differences in bacterial composition, however with small effect sizes, accounting for only 4 and 1% of variation, respectively, (Supplementary Figure 3C). Following pairwise testing, bacterial composition was distinct at 6 weeks from prior measurements (*p* < 0.05). Analysis of composition of microbiota with bias correction (ANCOM-BC) was used to identify significantly discriminating species between time points within each product group. The differential bacterial species are listed in Supplementary Tables S1, S2. Representative discriminant bacterial species are shown in Figure 2A. In the Prebiotic group, the pathogenic bacteria *P. stutzeri* was significantly decreased at 3 and 6 weeks and *S. anadarae* was reduced at 6 weeks compared to baseline with no change in the control group for either bacteria. Moreover, the relative abundance of *P. stutzeri* was significantly lower in the Prebiotic group vs. control at 6 weeks. Conversely, the commensal bacteria, such as *S. equorum* and *S. mitis,* increased at 3 and/or 6 weeks compared to baseline in the Prebiotic group, not in the control group.

### Functional pathways of skin microbiome by shotgun metagenomics

Alpha and beta diversity of metagenomic data were assessed using taxonomic abundances at strain level. No significant difference was observed between the groups, indicating no apparent microbial composition change. Two bacterial strains were identified as the discriminant bacteria between the groups. *Lactobacillus fermentum* ATCC 14931 was significantly decreased at 6 weeks in the control group, and not changed in the Prebiotic group. While *Halomonas desiderata* SP1 was significantly increased at 6 weeks in the Prebiotic group, but not changed in the control group (Figure 2B).

**Figure 3:**
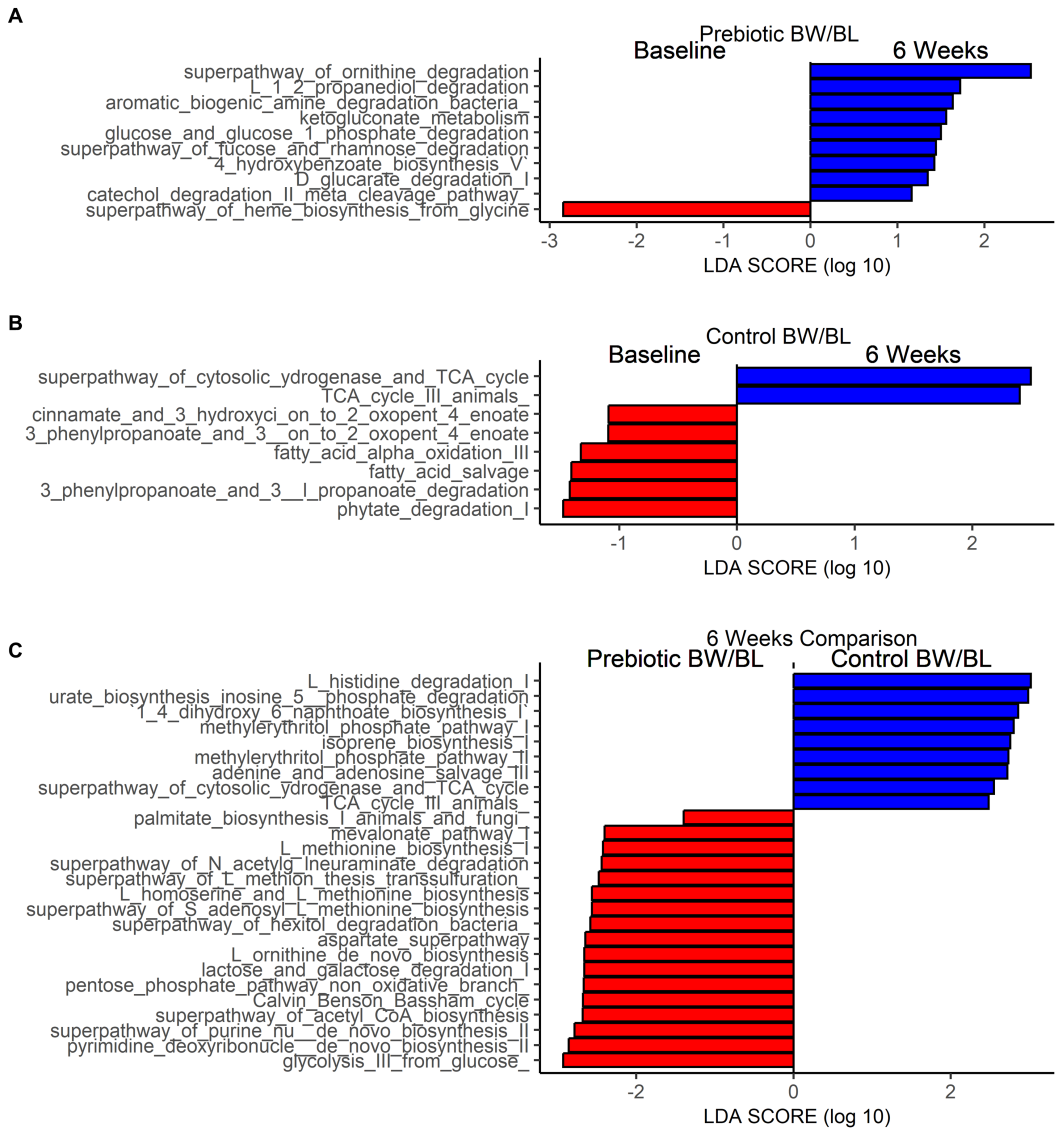
Functional characterization of skin microbiome by shotgun metagenomics. Differentially abundant bacterial MetaCyc pathways between baseline and after product application in Prebiotic Body wash (BW)/Body lotion (BL) group **(A)**, Control group **(B)** and between Prebiotic BW/BL and control group at 6 weeks **(C)** identified by Linear Discriminant Analysis Effect Size analysis (LEFSe). LEfSe is calculated with a Kruskal–Wallis alpha value of 0.05, a Wilcoxon alpha value of 0.05, and a logarithmic LDA score threshold of 1.0. Red bars to the left convey that the pathways in that group are more abundant in the “red” group than the other. Blue bars to the right convey that the pathways are more abundant in the “blue” group.

Linear Discriminant Analysis Effect Size (LEFSe) analysis was performed to identify MetaCyc pathways that are significantly different between time points within each group and between groups at 6 weeks (Figure 3). Based on the threshold value Linear Discriminant Analysis score (LDA) > 1.0 and *p* < 0.05, 9 MetaCyc pathways were significantly enriched at 6 weeks in the Prebiotic group compared to baseline (Figure 3A), including Amine and Polyamine Degradation pathways (superpathway_of_ornithine_degradation, aromatic_biogenic_amine_degradation_bacteria), sugar and sugar acids degradation pathways (glucose_and_glucose_1_phosphate_degradation, superpathway_of_fucose_and_rhamnose_degradation, ketogluconate_metabolism, D_glucarate_degradation_I), and Aromatic Compound Biosynthesis pathways (4_hydroxybenzoate_biosynthesis_V, catechol_degradation_II_meta_cleavage_pathway; Figure 2A). In contrast, more metabolic pathways were significantly depleted at 6 weeks in Control group (Figure 3B), including Fatty Acid and Lipid Biosynthesis (fatty_acid_salvage, fatty_acid_alpha_oxidation_III), Aromatic Compound Degradation (cinnamate_and_3_hydroxyci_on_to_2_oxopent_4_enoate, ‘3_phenylpropanoate_and_3__on_to_2_oxopent_4_enoate), Sugar Derivative Degradation (phytate_degradation_I) and Aromatic Compound Biosynthesis pathways. Comparing the two groups at 6 weeks, Fatty Acid and Lipid Biosynthesis, Amino acid Biosynthesis, Carbohydrate Degradation, Nucleotides Biosynthesis pathways are more enriched in the Prebiotic group. Secondary metabolites Biosynthesis and Amino acid Degradation pathways are more enriched in the control group (Figure 3C).

### Metabolic profiles on the skin surface by metabolomics

**Figure 4:**
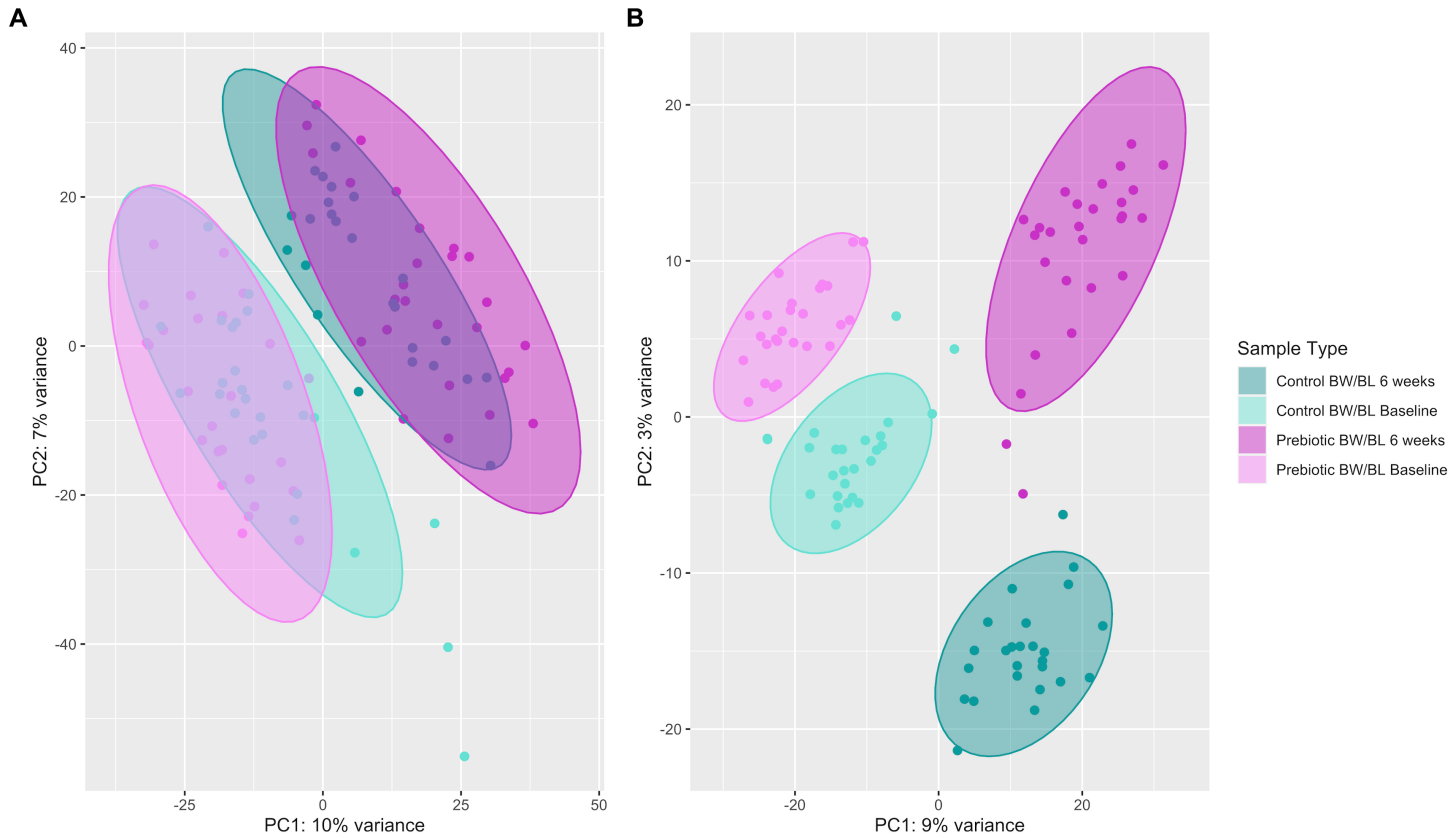
Principal component analysis (PCA) plot **(A)** and Partial least squares-discriminant analysis (PLS-DA) plot using 100 random permutations and 5-fold cross-validation **(B)** of skin metabolic profiles characterized by LC–MS/MS Neg using the R package ropls v1.24.0. Confidence ellipse level at 95%. BW, body wash; BL, body lotion.

Untargeted skin metabolomic profiles were characterized by LC–MS/MS reserve phase (RP) using negative and positive ion modes. 3,508 metabolic features were detected by LC–MS/MS Neg data after background filtering. Principal component analysis (PCA) shows clear cluster separation between the samples from baseline, 6 weeks and skincare products (Supplementary Figure S4). After removing skincare products’ samples, the separation between the product groups remains significant (Figure 4A). This separation is further confirmed using Partial least squares-discriminant analysis (PLS-DA; *n* = 100, R2Y = 0.98, *p* (R2Y) = 0.01, Q2 = 0.85, *p* (Q2) = 0.01; Figure 4B), indicating that the skin metabolic profiles were significantly changed by the products. And the changes in skin metabolome seem more apparent compared to microbiome data (Figure 4A; Supplementary Figure S3C).

1,209 metabolite features were detected as discriminating metabolites between the time points within each product group (Variable Importance in Project (VIP) > 1 and Wilcoxon Rank Sum Test *q*-value <0.05). 96 of 1,209 metabolites had moderate and significant correlation to skin hydration (Spearman’s correlation |*ρ*| > 0.4 and *q*-value <0.05). No metabolite had significant correlation to TEWL. Supplementary Table S3 lists all 96 clinically relevant metabolite features/classes and the effect size (Cohen’s d) in each product group (annotation, if given, is at the level 2–3). For the majority of the metabolites (70 of 96), the Prebiotic group had a higher effect size (bigger change between Baseline and 6 weeks) than the control group, including long-chain/medium-chain fatty acids, Fatty acid esters, Fatty Acyls and Dicarboxylic acids and derivatives.

The results of LC–MS/MS Pos were described in Supplementary material. Briefly, the PCA and PLS-DA analysis of LC–MS/MS Pos data showed significant separation between the groups (Supplementary Figure S5). The Prebiotic group had a higher effect size for most of the discriminant clinically relevant metabolite features (50 of 90) than the control group, such as monosaccharides (Supplementary Table S4).

Both LC–MS/MS Neg and Pos data indicated that the Prebiotic group had larger modulation on many clinically relevant metabolites compared to the control group.

### Correlation of skin bacteria, metabolites and clinical outcomes

**Figure 5:**
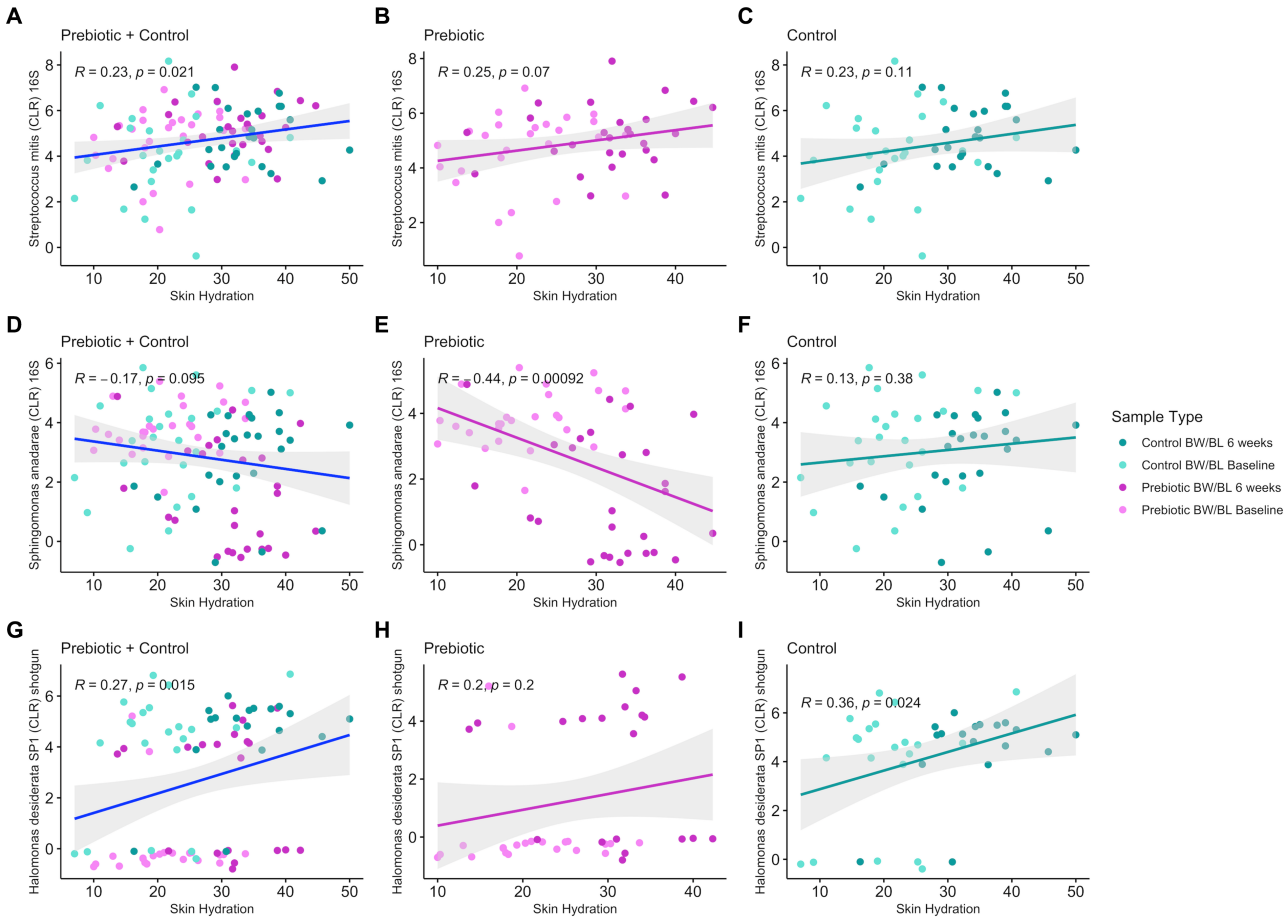
Pearson’s correlation between discriminant bacteria identified from 16S rRNA gene sequencing **(A–C)** and shotgun metagenomics **(D–I)** and Skin Hydration in all treatment groups using the R package ggpubr v.0.6.0 and 95% confidence level with linear regression.

We employed an integrated multi-omics approach to investigate the relationship between the metabolome, microbiome, and clinical outcomes in this study. Initially, PLS-DA was conducted to understand the global metabolome shifts across different time points and treatments. Subsequently, discriminating metabolites were identified based on their significant contributions to sample differentiation. These metabolites were then correlated with clinical measurements using Spearman correlation analysis, focusing on Skin Hydration and Skin TEWL, yielding 96 discriminant clinically relevant metabolites. The 96 discriminant metabolites were further correlated with microbial composition/functional pathways (16S rRNA, shotgun sequencing, MetaCyc pathways) using Spearman correlation. Given the weak separation between groups for microbiome data, Spearman’s correlation cutoff was set to |*ρ*| = 0.3 (moderate) for microbiome-metabolome correlations, however there were no additional significant correlations below |*ρ*| = 0.3. Given the compositional nature of microbiome data, microbial counts were transformed into a log-ratio space using centered log-ratio (CLR) transform. For 16S rRNA sequencing, there were 27 unique microbes, including *S. anadarae* and *P. stutzeri*, previously identified as discriminant bacteria between groups (Figure 2), having at least one significant correlation to 32 clinically relevant metabolite features (Spearman’s correlation |*ρ*| > 0.3 and *q*-value <0.05) including fatty acids, Dicarboxylic acids. For metagenomic data, only strain *H. desiderata* SP1 had a significant correlation to two clinically relevant metabolites. Additionally, *S. mitis* (*R* = 0.23, value of *p* = 0.021) and *H. desiderata* SP1 (*R* = 0.28, value of *p* = 0.01) had a positive correlation to skin hydration, while *S. anadarae* (*R* = −0.17, value of *p* = 0.095) tended to negatively correlate to skin hydration (Figure 5). Figures 5A,D,G show the overall relationship between the abundance of specific microbes and skin hydration across all treatment groups. The significant correlation between microbial abundance and skin hydration (for *S. mitis* and *H. desiderata SP1* specifically) suggests that the microbial abundance may play a role in determining skin hydration but the strength of the association may be modulated by treatment. Figures 5B,C,E,F,H,I compare the strength and direction of the correlation within each treatment group. We observed that discriminant metabolites from both LC–MS/MS Neg and Pos data are not only significantly correlated with *S. anadarae* and *P. stutzeri* but with multiple species from the same genera whilst sharing similar patterns of correlation (e.g., *S. gimensis*, *S. hunanensis*, *S. japonica*, *P. aeruginosa* and *P. fluorescens*).

**Figure 6:**
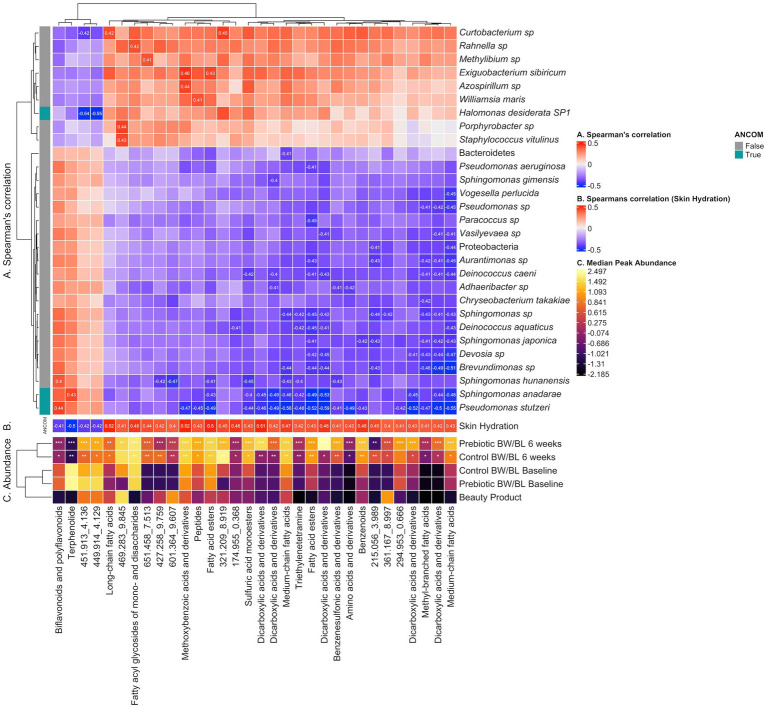
Heatmap illustrating correlation between microbes, clinical outcomes and discriminant metabolite features identified by LC–MS/MS Neg. **(A)** Spearman’s correlation between discriminant metabolite features and microbes, computed using the rcorr function from the R package Hmisc v.5.0.1 and *p*-values adjusted using the Benjamini and Hochberg correction. Correlations having rho >0.3 (low correlation and above) and q-value <0.05 are labeled. **(B)** Pearson’s correlation between discriminant metabolite features and Skin Hydration. Correlations having q-value <0.05 are labeled. **(C)** Median peak abundance (normalized, log transformed) per discriminant metabolite feature within each treatment group. Differential abundance significance between Baseline vs. 6 weeks per Control or Prebiotic group is illustrated using * for *q*-value <0.05, ** for *q*-value <0.01 and *** for *q*-value <0.001 (paired Wilcoxon Rank Sum Test, Benjamini & Hochberg adjusted). Cells in the Beauty Product row labeled “P” (Prebiotic) or “C” (Control) suggest the corresponding metabolite at 6 weeks originates from the beauty product. BW, body wash; BL, body lotion.

No bacteria had correlation with TEWL. MetaCyc pathways and clinically relevant metabolites yielded no significant correlations. Notable examples of microbe-metabolite correlations are illustrated in Figure 6.

The correlation analysis between microbiome and discriminant clinically relevant metabolites from LC–MS/MS Pos was described in Supplementary material. Notable examples of microbe-metabolite correlations are illustrated in Supplementary Figure S6.

## Discussion

The benefit of the triple-biotics has been demonstrated in the previous study (10). Here we further validated the prebiotic effect of the body wash and body lotion containing triple-biotics in a clinical study. Even though the overall microbial composition was not significantly changed, we did observe relatively small apparent changes in bacterial taxonomic composition and function, which were coupled to surprisingly dramatic shifts in skin metabolome, suggesting even minor shifts in microbial abundance can lead to a significant modulatory effect.

Certain potential pathogens, such as *P. stutzeri* and *S. anadarae* were significantly reduced in the Prebiotic group*. P. stutzeri* is an opportunistic pathogenic bacteria, which has been shown to cause skin infections (32). The role of *S. anadarae* on skin health is not well studied, however, a few studies have shown other *Sphingomonas* species, such as *S. paucimobilis* (previously known as *Pseudomonas paucimobilis*) (33) is an occasional human pathogen causing infection (34). Moreover, we discovered that *S. anadarae* tended to negatively correlate with skin hydration, suggesting that this genus may have a negative impact on skin health. The prebiotic BW/BL also significantly increased *S. equorum, S. mitis* and *H. desiderata SP1*, which are commensal bacteria on the skin surface (1, 35). Additionally, the increase of *S. mitis* and *H. desiderata SP1* are positively correlated with skin hydration. They could be used as potential targets in developing products or solutions to improve skin health. However, the role of these bacteria, especially *S. anadarae* and *H. desiderata SP1* remains to be investigated. It appears that the prebiotic BW/BL has a positive effect on skin microbiome composition mainly due to the triple-biotics, as inulin is a well-known prebiotics to support beneficial bacteria, and butyloctanol has been shown to target undesirable bacteria more effectively than beneficial bacteria (10).

The prebiotic BW/BL also elevated the bacterial metabolic pathways, especially sugar/sugar acid degradation pathways compared to baseline. Additionally, lactose and galactose degradation pathways were significantly increased in the Prebiotic group compared to Control at 6 weeks (Figures 3A,C). The activation of carbohydrate degradation metabolism could be largely due to inulin. Inulin is a polysaccharide and can only be utilized by bacteria to generate short chain fatty acids, and other carbonic acids such as lactic acid (36). Lactic acid is a key component of the skin’s natural moisturizing factor and keeps skin hydrated (37). It also affects skin pH and is beneficial to epidermal turnover, and thus is used as an ingredient in various skin care products (38). In this study, we demonstrated the benefit of topically applied inulin on skin hydration and the MOA through modulation of bacterial carbohydrate metabolism. In contrast, the control group reduced the bacterial metabolic pathways, esp. fatty acid biosynthesis pathways (Figure 3B). Fatty acids are the most important components in maintaining the skin barrier function (39). Reduction of fatty acid biosynthesis pathways may have a negative impact on skin health. In our study, we found that prebiotic BW/BL showed a positive effect on skin microbiome structure and activity leading to the increased skin hydration, while the increase of skin hydration in the control group may be due to the other ingredients, such as humectants.

The skin metabolome showed more apparent changes by the products compared to the microbiome data. The discriminant metabolites among the groups were also found to correlate with skin hydration. The prebiotic group had a greater modulation on many clinically relevant metabolites including long-chain/medium-chain fatty acids, Fatty acid esters, Fatty Acyls, Dicarboxylic acids and derivatives. These metabolites are known to have beneficial effects on skin health. For instance, fatty acids and esters, fatty acyls are the components of skin lipids contributing to skin barrier functions (39, 40). Dicarboxylic acids have antimicrobial and anti-inflammatory properties, which have been widely used in skincare products to offer skin benefits (41). Additionally, monosaccharides detected by LC–MS/MS Pos were higher in the Prebiotic group, which may result from the fermentation of inulin by skin bacteria. This result is also consistent with the metagenomic data showing carbohydrate degradation metabolism was activated in the Prebiotic group.

A significant correlation between skin microbiome and multiple metabolites was observed. The reduction of *S. anadarae* and *P. stutzeri* were negatively correlated with many metabolites including fatty acids and Dicarboxylic acids, while the increase of *H. desiderata* SP1 had positive correlation to certain metabolites. Moreover, many of the same metabolites are also found to positively correlate with the increase of skin hydration. The findings implied that the changes in abundances of these specific bacteria and their associated metabolites may contribute to the increase of skin hydration.

In summary, this study revealed an apparent beneficial effect of topically applied pre/postbiotics on skin microbiome and corresponding metabolome. The concurrent changes in microbiome and metabolome were correlated with the clinical outcomes, especially skin hydration. Therefore, we hypothesize that the products containing the pre/postbiotics benefit skin health through the MOA of modulation of skin microbial composition towards taxa with greater functional capacity to produce metabolites that may link to the increase of skin hydration. This study provides insights into the underlying mechanism of topical pre/postbiotics on skin health. The bacteria and metabolites that were identified to be associated with skin hydration could be potentially used as targets in developing innovative topical treatments to manage skin health.

## Data availability statement

The datasets presented in this study can be found in online repositories. The names of the repository/repositories and accession number(s) can be found at: https://www.ncbi.nlm.nih.gov/, PRJNA902366, https://massive.ucsd.edu, https://gnps.ucsd.edu, MSV000090708.

## Ethics statement

The studies involving human participants were reviewed and approved by Reinhold Gahlmann, Peter Dolfen, Friedrich-Peter Vollmer, Thomas Mertens-Ammermann, Daniel Kiwitt. The patients/participants provided their written informed consent to participate in this study.

## Author contributions

ML, ID, NS, AMM, TB, and JM: conceptualization. ML and ID: data curation. ML, ID, EK, AVM, AA, CT, and JM: formal analysis and writing original draft preparation. AMM and TB: funding acquisition. ID, AMM, TB, and JM: investigations. ML, ID, NS, EK, AVM, AA, and JM: methodology. ID, AMM, TB, and JM: supervision. NS, AMM, TB, JM, and AA: writing – review and editing. All authors contributed to the article and approved the submitted version.

## Funding

The project was funded by Colgate-Palmolive.

## Conflict of interest

This study received funding from Colgate-Palmolive. The funder had the following involvement with the study: clinical study design, multi-omics data analysis and interpretation, decision to publish and preparation of the manuscript. ML, ID, NS, AMM, TB, and JM are employees of Colgate-Palmolive. AA, AVM, and EK are consultants of Clarity Genomics, Inc., and Arome Science Inc., and were compensated by Colgate-Palmolive for their contributions to the mass spectrometry data acquisition and analysis. CT is the employee of RTL Genomics and was compensated by Colgate-Palmolive for the contribution to 16S sequencing analysis.

## Publisher’s note

All claims expressed in this article are solely those of the authors and do not necessarily represent those of their affiliated organizations, or those of the publisher, the editors and the reviewers. Any product that may be evaluated in this article, or claim that may be made by its manufacturer, is not guaranteed or endorsed by the publisher.
