# Supplementary material for: Multi-omic approach to decipher the impact of skincare products with pre/postbiotics on skin microbiome and metabolome
Source: Front Med (Lausanne). 2023 Jul 18;10:1165980. doi: 10.3389/fmed.2023.1165980 (PMC10392128; doi:10.3389/fmed.2023.1165980)
Supplement: Supplementary file 1 [file Data_Sheet_1.docx]

Supplementary Material

**Multi-omic approach to decipher the impact of skincare products with pre/postbiotics on skin microbiome and metabolome**

Min Li^1^, Junhong Mao^1*^, Isabel Diaz^1^, Evguenia Kopylova^2,3^, Alexey V. Melnik^2,3^, Alexander A. Aksenov^2,3^, Craig D. Tipton^4^, Nadia Soliman^1^, Andrea M. Morgan^1^, Thomas Boyd^1^

^1^ Colgate−Palmolive Company, Piscataway, New Jersey, United States

^2^ Clarity Genomics Inc., San Diego, California, United States

^3^ Arome Science Inc., Farmington, Connecticut, United States

^4^ RTL Genomics, MicroGenDX, Lubbock, Texas, United States

*corresponding author: Junhong Mao

junhong_mao@colpal.com

**SUPPLEMENTARY MATERIALS AND METHODS**

**Clinical study design**

A randomized clinical study was conducted by ProDERM (Schenefeld, Germany) to assess the impact of skincare products with pre/postbiotics on the skin microbiome and metabolome of normal and dry to extremely dry skin. Eighty-four female subjects (18-70 yrs) with normal and dry to extremely dry skin on the lower legs were enrolled according to self-estimation and visual assessment by a dermatologist. Eighty-one subjects were analyzed, 2 subjects dropped out and 1 subject demonstrated protocol deviations and was excluded. During the wash-out phase, all subjects were assigned to use a standard marketed shower gel (Palmolive®, Colgate-Palmolive, Piscataway, New Jersey) once daily on their body, except for their legs (subjects were instructed to rinse with water only) for 7 days. There were a total of three test groups (1 normal skin group and 2 dry/extremely dry skin groups). One dry/extremely dry skin group (N=27) and the normal skin group (N=28) were given a body wash and body lotion with triple-biotics (Sanex®, Colgate-Palmolive). The other dry/extremely dry skin group (N=26) continued to use the washout shower gel and a body lotion (E45 Daily Lotion, Reckitt-Benckiser, [Slough, U](https://www.google.com/search?sxsrf=APq-WBtPVGCMfhLkqrdhqN49xT8oz7o3iA:1650995012557&q=Slough&stick=H4sIAAAAAAAAAOPgE-LUz9U3MEvPs8xS4gAxky2Ty7W0spOt9POL0hPzMqsSSzLz81A4VhmpiSmFpYlFJalFxYtY2YJz8kvTM3awMu5iZ-JgAACuEkDBVAAAAA&sa=X&ved=2ahUKEwjr-f7Zo7L3AhVEU98KHe3JBeQQmxMoAXoECGMQAw)K). The subjects washed with the body wash once daily in week 1 to 6, and applied the body lotion twice daily in weeks 4 to 6 on their body. During the study, the subjects were not allowed to use any other skin care products on their lower legs.

At baseline, 3 and 6 weeks after product application, skin hydration was measured by Corneometer CM 825 (Courage & Khazaka, Cologne, Germany). Transepidermal water loss (TEWL) was measured by Tewameter® TM 300 (Courage & Khazaka, Cologne, Germany). One skin swab was taken at each assessment time for 16S rRNA sequencing from the lower leg for all three test groups. Two swabs were collected from the lower leg at baseline and 6 weeks for shotgun metagenomic sequencing and metabolomics analysis, respectively for the two dry/extremely dry skin groups.

**16S rRNA gene sequencing data analysis**

V1-3 hypervariable region of 16S rRNA gene sequencing was conducted by RTL Genomics (Lubbock, Texas). DNA was extracted via KingFisher FLEX instrument (ThermoFisher Scientific, Inc., Waltham, Massachusetts) and using Zymo ZR-96 magbead kit (Zymo Research, Irvine, California) following manufacturer’s instructions. The extraction protocol was modified to include a mechanical lysis step with a Qiagen TissueLyser. V1-3 regions of 16S rRNA gene were amplified for sequencing in two-step, independent reactions using HotStar Taq Master Mix Kit (Qiagen) with 28F-519R primers (28F: 5”GAG TTT GAT CNT GGC TCA G 3”; 519R: 5” GTN TTA CNG CGG CKG CTG 3”). PCR amplification included 0.5 μl of 5 μM forward primer, 0.5 μl of 5 mM reverse primer, 5 μl of DNA template, and 14 μl of Taq Master Mix. To encourage amplification in low biomass samples, 2 ul BSA and 2 ul MgCl2 were added to reactions. The negative control was a reaction mixture with no template DNA. PCR reaction conditions included initial denaturation at 95○C for 5 min, then 10 cycles of 94○C for 30 s, 50○C for 90 s (+0.5○C per cycle), 72○C for 1 min, followed by 25 cycles of 94○C for 30 s, 54○C for 90 s, 72○C for 1 min, and finally, one cycle of 72○C for 10 min and 4○C hold. Barcoding PCR reactions conditions included initial denaturation at 95○C for 5 min, then 10 cycles of 94○C for 30 s, 54○C for 40 s, 72○C for 1 min, followed by one cycle of 72○C for 10 min and 4○C hold. Amplification products were visualized with eGels (Life Technologies). Products were then pooled equimolar and each pool was size selected in two rounds using SPRIselect beads (BeckmanCoulter) in a 0.75 ratio for both rounds. Size selected pools were then quantified using Qubit 4 fluorometer (Life Technologies) and loaded on an Illumina MiSeq 2x300 flow cell at 10 pM for sequencing. The sequences were clustered into OTUs at 97% sequence similarity. Denoising of sequence reads, chimera detection, and stitching of 2x300 paired reads were conducted using Usearch7 (Edgar et al., 2010), UCHIME (Edgar et al., 2011) cite, and PEAR (Zhang et al., 2014), respectively. Quality filtered and assembled reads were clustered into OTUs at 97% sequence similarity threshold using the UPARSE algorithm. OTU assignment then used the RTL Genomics in-house taxonomic reference database. Multiple sequence alignment and phylogenetic tree estimation of representative OTU sequences was performed for downstream analysis using MUSCLE (Edgar, 2004) and FastTree2 (Price et al., 2010).

Eight extraction blanks were sequenced, and obtained a total of ~43k read counts (0.7% of total reads) and 52 OTUs were identified which may be contaminants, based at least 2X disproportionately greater detection in extraction controls than true samples. Overall, mapping possible contaminant OTUs to samples indicated that ~3.8% of true sample read counts are composed of OTUs which may be contaminants. Based on these reasons: (a) the OTUs composed a negligible percentage of read counts, (b) included taxa frequently reported in the human microbiome, and (c) removing all OTUs found matching with controls may remove true biological sequences especially from well-to-well splash over (source/sink dynamics unclear), we did not remove all identified possible contaminants from the following analysis. However, one common reagent contaminant genus, *Pelomonas*, was removed per lab recommendation.

Bacterial alpha and beta diversity were summarized by Shannon diversity and by weighted UniFrac distance (Lozupone et al., 2011), respectively. Product group and time point were each investigated for their influence on alpha and beta diversity, using ANOVA and PERMANOVA, as implemented in the R function adonis. Principal coordinates analysis (PCoA) was used to visualize compositional similarity among microbiome communities. To evaluate differences in the relative abundances of taxa between cohorts, ANCOM-BC (Lin et al., 2020) procedure was carried out on taxa present in at least 20% of samples, using the arguments struc zero = T and neg lb = T. Holm’s method was used to adjust p-values and account for multiple test correction. p-value <0.05 is considered statistically significant.

**Shotgun Metagenomic sequencing and data analysis**

Shotgun metagenomic sequencing was conducted by CosmosID (Germantown, Maryland). 20uL of Zymo Spike-In 2 was added to the skin swabs which were extracted with QIAGEN PowerSoil Pro, according to the manufacturer's protocol. DNA libraries were prepared using the Nextera XT DNA Library Preparation Kit (Illumina). Genomic DNA was fragmented using a proportional amount of Illumina Nextera XT fragmentation enzyme. Unique dual indexes were added to each sample followed by 12 cycles of PCR to construct libraries. DNA libraries were purified using AMpure magnetic Beads (Beckman Coulter, [Brea, C](https://www.google.com/search?sxsrf=ALiCzsahuGCMHE8WT-6KDCyvXjIf68jD0A:1651594909222&q=Brea,+California&stick=H4sIAAAAAAAAAOPgE-LSz9U3MC4wzClOV-IAsYuMkgy0tLKTrfTzi9IT8zKrEksy8_NQOFYZqYkphaWJRSWpRcWLWAWcilITdRScE3My0_KL8jITd7Ay7mJn4mAAAIkqz-9fAAAA&sa=X&ved=2ahUKEwi65IG_3sP3AhVFmeAKHVK8B2kQmxMoAXoECG8QAw)alifornia) and eluted in QIAGEN EB buffer. DNA libraries were quantified using Qubit 4 fluorometer and Qubit™ dsDNA HS Assay Kit. Libraries were then sequenced on an Illumina NovaSeq S4 platform 2x150bp.

Shotgun Metagenomics Taxonomic Classification Methods:

The system utilizes a high performance data-mining k-mer algorithm that rapidly disambiguates millions of short sequence reads into the discrete genomes engendering the particular sequences. The pipeline has two separable comparators: the first consists of a pre-computation phase for reference databases and the second is a per-sample computation. The input to the pre-computation phase is the databases of reference genomes that are continuously curated by CosmosID scientists. The output of the pre-computational phase is a phylogeny tree of microbes, together with sets of variable length k-mer fingerprints (biomarkers) uniquely associated with distinct branches and leaves of the tree. The second per-sample computational phase searches the hundreds of millions of short sequence reads, or alternatively contigs from draft de novo assemblies, against the fingerprint sets. This query enables the sensitive yet highly precise detection and taxonomic classification of microbial NGS reads. The resulting statistics are analyzed to return the fine-grain taxonomic and relative abundance estimates for the microbial NGS datasets. To exclude false positive identifications the results are filtered using a filtering threshold derived based on internal statistical scores that are determined by analyzing a large number of diverse metagenomes.

Shannon alpha diversity was calculated from the strain-level abundance score matrices using the R package Vegan v2.6-4. Wilcoxon Rank-Sum tests were performed between groups. Beta diversity Principal Coordinate Analyses were calculated based on strain matrices. Bray-Curtis dissimilarity was calculated in R using vegan with the function vegdist, and PCoA tables were generated using ape v5.6-2 (Paradis et al., 2004) function pcoa. PERMANOVA test was performed for each distance matrix using vegan’s function adonis2, and beta dispersion was calculated and compared using the anova method for the betadisper function from vegan.

Shotgun Metagenomics Functional Classification Methods

Initial QC, adapter trimming and preprocessing of metagenomic sequencing reads are done using BBduk (<https://sourceforge.net/projects/bbmap/>). The quality controlled reads are then subjected to a translated search against a comprehensive and non-redundant protein sequence database, UniRef 90. The UniRef90 database, provided by UniProt ([The UniProt Consortium](https://pubmed.ncbi.nlm.nih.gov/?term=UniProt%C2%A0Consortium%20T%5BAuthor%5D) 2018), represents a clustering of all non-redundant protein sequences in UniProt, such that each sequence in a cluster aligns with 90% identity and 80% coverage of the longest sequence in the cluster. The mapping of metagenomic reads to gene sequences are weighted by mapping quality, coverage and gene sequence length to estimate community wide weighted gene family abundances as described by Franzosa et al (Franzosa et al., 2018). Gene families are then annotated to MetaCyc ([Caspi](https://pubmed.ncbi.nlm.nih.gov/?term=Caspi%20R%5BAuthor%5D) ET AL., 2016) reactions (Metabolic Enzymes) to reconstruct and quantify MetaCyc metabolic pathways in the community as described by Franzosa et al (Franzosa et al., 2018). To facilitate comparisons across multiple samples with different sequencing depths, the abundance values are normalized using Total-sum scaling (TSS) normalization to produce "Copies per million" (analogous to TPMs in RNA-Seq) units.

**Mass spectrometry-based metabolomics and data analysis**

Skin sample preparation for LC-MS analysis

Skin swabs were spiked with 5μl of Short chain fatty acids (Fisher) and Lactate (Fisher) mixture (2mM). For skin products, 5μl of product was placed in an Eppendorf tube, weighed and diluted with 1 ml of 50% methanol. The 5μl of solution was deposited on swabs. Beauty products containing swabs were extracted together with all samples. Tubes with skin swabs samples were placed on ice to keep them at 0-4 ○C and 300μl of the Extraction Solvent (LC-MS water + mix of internal standards 5C13-Glu (Sigma-Aldrich) and sulfachloropyridazine at 50μM) was added to each tube. In parallel, 2 process extraction solvent blanks and swab blanks were prepared and processed with all other samples. All samples were sonicated for 5 minutes on iced water, spinned down for 2 minutes. 200μl of the methanol was then added to all tubes containing swabs and the soaked cotton buds were extracted at 4°C for 12 hours. Each cotton bud was removed with a pair of tweezers, and as much solvent as possible was retrieved by pressing the bud against the side of the well. Each tube solvent was evaporated using the bench top vacuum centrifuge concentrator (450 g-force) until dry then was re-dissolved in 100μl of 2:8 (vol/vol) methanol: water at room temperature. Tubes were agitated gently and placed it in an ultrasonic bath for 5 min to ensure appropriate dissolution of the sample then centrifuged at 450 g for 10 minutes at room temperature; 80μl of the tube content was transferred into a glass vial using a pipette. To create a pooled quality control (QC) sample, 5μl from each of all sample tubes were added together into a new micro centrifuge tube. The resultant samples were then analyzed using LC-MS as described below.

Liquid Chromatography - Mass Spectrometry (LC-MS) data analysis, reverse phase (RP)

For the RP LC analysis, Thermo Orbitrap instrument interfaced with a Waters BEH C18 (100mm x 2.1 mm x 1.7uM) column powered by Vanquish LC system was used. Samples were maintained at 4°C throughout the analysis. Data were acquired using DDA mode in positive and negative modes. Aqueous mobile phase (Solvent A) for LC-MS was prepared by adding LC-MS grade formic acid to LC-MS grade water to make a 100:0.1 (vol/vol) water: formic acid mix. Organic LC-MS mobile phase (Solvent B) was prepared by adding LC-MS grade formic acid to LC-MS grade acetonitrile to make a 100:0.1 (vol/vol) acetonitrile: formic acid mix. The LC gradient was as follows: Time 0 min - 5%B; 5 min - 95%B; 7 min - 95%B; 7.1 min - 5%B; 8 min - 5%B, at flow of 0.4 ml/min. Column compartment was held at 30°C using forced air. The MS parameters were as follows: Normalized collision Energy: 30 eV; CID Isolation width: 1 m/z; Mass range 70-1050 m/z. ESI source settings: Spray voltage: 3.5 k; Source heater temp: 350 C; Sheath gas flow rate: 50 ml/min; Aux gas flow rate: 7 ml/min; Capillary temp: 350 C; Spray voltage: 3.5 kV. Product ion spectra were recorded in data dependent acquisition (DDA) mode with MS1 full scan at 100-1200 m/z at the 17,500 resolution and up to 5 MS/MS scans (TOP5) of the most abundant ions per duty cycle with the 17,500 resolution with 1 microscan in either positive or negative mode. Normalized collision energy was set to stepwise increase from 20 to 30 to 40 units with z = 1 as default charge state. MS/MS experiments were set to be automatically triggered at the apex of a peak within 2 to 15 s from their first occurrence with the Dynamic exclusion time was set to 5 s.

Liquid Chromatography - Mass Spectrometry (LC-MS) data processing

The data were converted from vendor’s to mzXML format. The feature detection was then carried out using MZmine2 ([Pluskal](https://pubmed.ncbi.nlm.nih.gov/?term=Pluskal+T&cauthor_id=20650010) et al., 2010) with the following settings: feature extraction signal threshold of 5.0E3; minimum peak width 3 sec. According to the statistical evaluation of the quality control (QC) samples the mass tolerance was set to 5 ppm and maximum allowed retention time deviation to 10 sec. For the chromatographic deconvolution maximum peak width was set to 2 min. After the removal of isotope peaks, the peak lists were aligned with the above-mentioned retention time and mass tolerances. The Arome Science’s online workflow for feature-based molecular networking (Nothias et al., 2020) was then used to create molecular networks and annotate features by searching against GNPS public and commercial NIST 2020 libraries.

Metabolomics data analysis

Using the MetaboAnalyst R package (version 3.1.0), zero values were replaced by half of the smallest positive value in the original metabolomics feature table and normalized to an internal standard (Sulfachloropyridazine). Background noise filtering was performed to retain features having a minimum 3x peak abundance in at least one clinical sample compared to the blank samples. For PCA and PLS-DA analysis, data was mean-centered, unit variance scaled and log10 transformed. Feature outlier detection and PLS-DA analysis were performed using the ROBPCA algorithm (Hubert et al., 2015) from the ropls R package (version 1.24.0). PLS-DA models were tested at 100 permutations with 5-fold cross validation. Univariate analysis was performed using the paired Wilcoxon Rank Sum test between time points within each product group and unpaired Wilcoxon Rank Sum test between clinical samples and skincare product samples. Discriminating metabolites separating time points and product groups were identified as those having VIP value equal to or greater than 1 and Wilcoxon Rank Sum test FDR-adjusted q-value < 0.05. In untargeted metabolomics and shotgun metagenomics, the distribution of metabolite intensities or read sequence counts can be skewed making it challenging to satisfy the assumptions of parametric tests, thus the Wilcoxon Rank Sum test was used for univariate tests.

Correlation of discriminating metabolites with clinical data (skin hydration and skin TEWL) as well as 16S, shotgun sequencing species and pathways was assessed using Spearman’s correlation (*rcorr* function from the “Hmisc” R package), as this metric evaluates monotonic relationships between variables (and microbe-metabolite relationships are often complex and may not follow a linear pattern, as well as often exhibiting non-normal distributions with skewed or heavy-tailed data), and p-values adjusted using the Benjamini and Hochberg correction using the *p.adjust* function from the “stats” R package.

**SUPPLEMENTARY RESULTS**

3136 metabolic features were detected by LC-MS/MS Pos after background filtering. The PCA and PLS-DA analysis of LC-MS/MS Pos data also showed significant separation between the groups (Supplementary Figure 5). 1054 metabolite features were detected as discriminating metabolites between the time points within each product group. 87 and 3 of 1054 metabolites had moderate and significant correlation to skin hydration and skin TEWL (|*p*| > 0.4 and q-value < 0.05), respectively. The effect size for each discriminating metabolic feature and product group was shown in the Supplementary Table 4 (annotation, if given, is at the level 2-3). 52 of 90 metabolic features had a larger effect size for the Prebiotic group, including Phenol ethers, Benzenoids, Monosaccharides, Fatty acyl glycosides of mono- and disaccharides. Some of the metabolic features had a larger effect size control group including Lineolic acids and derivatives, Amino acids and Glycerolipids.

Correlation analysis was performed between microbiome composition/functional pathways (16S, shotgun sequencing, MetaCyc pathways) and 90 discriminant clinically relevant metabolite features identified from LC-MS/MS Pos. For 16S sequencing data, there were 39 unique microbes including *S. anadarae* and *P. stutzeri* having Spearman’s correlation |ρ| > 0.3 and q-value < 0.05 to 40 (of 90) clinically relevant metabolite features. For shotgun sequencing data, no microbe or MetaCyc pathways had a significant correlation to clinically relevant metabolites. Notable examples of microbe-metabolite correlations are illustrated in Supplementary Figure 6.

**SUPPLEMENTARY REFERENCES**

Caspi, R., [Billington](https://pubmed.ncbi.nlm.nih.gov/?term=Billington%20R%5BAuthor%5D), R., [Ferrer](https://pubmed.ncbi.nlm.nih.gov/?term=Ferrer%20L%5BAuthor%5D), L., [Foerster](https://pubmed.ncbi.nlm.nih.gov/?term=Foerster%20H%5BAuthor%5D), H., [Fulcher](https://pubmed.ncbi.nlm.nih.gov/?term=Fulcher%20CA%5BAuthor%5D), C.A., [Keseler](https://pubmed.ncbi.nlm.nih.gov/?term=Keseler%20IM%5BAuthor%5D), I.M., et al. (2016). The MetaCyc Database of metabolic pathways and enzymes and the BioCyc collection of Pathway/Genome Databases. [Nucleic Acids Res.](https://www.ncbi.nlm.nih.gov/pmc/articles/PMC4702838/) 44(Database issue): D471–D480

Dührko, K., [Fleischauer](https://www.nature.com/articles/s41592-019-0344-8#auth-Markus-Fleischauer), M., Ludwig, M., [Aksenov](https://www.nature.com/articles/s41592-019-0344-8#auth-Alexander_A_-Aksenov), A.A., [Melnik](https://www.nature.com/articles/s41592-019-0344-8#auth-Alexey_V_-Melnik), A.V., [Meusel](https://www.nature.com/articles/s41592-019-0344-8#auth-Marvin-Meusel), M., et al. (2019). SIRIUS 4: a rapid tool for turning tandem mass spectra into metabolite structure information. Nat Methods. 16, 299-302.

Edgar, R.C., Haas, B.J., Clemente, J.C., Quince, C., Knight, R. (2011). UCHIME improves sensitivity and speed of chimera detection. Bioinformatics. 27, 2194–2200.

Edgar, R.C. (2004). MUSCLE: multiple sequence alignment with high accuracy and high throughput. Nucleic Acids Res. 32,1792-1797.

Edgar, R.C. (2010). Search and clustering orders of magnitude faster than BLAST. Bioinformatics. 26, 2460–2461.

Franzosa, E., McIver, L.J., Rahnavard, G., Thompson, L.R., Schirmer, M., Weingart, G., et al. (2018). Species-level functional profiling of metagenomes and metatranscriptomes. Nat Methods. 15, 962-968.

Hubert, M., Rousseeuw, P.J., Branden, K.V. (2005). ROBPCA: A New Approach to Robust Principal Component Analysis. Technometrics. 47, 64-79.

Lin, H., Peddada, S.D. (2020). Analysis of compositions of microbiomes with bias correction. Nat. Commun. 11, 3514.

Lozupone, C., Lladser, M.E., Knights, D., Stombaugh, J., Knight, R. (2011). UniFrac: an effective distance metric for microbial community comparison. ISME J. 5,169-172.

Nothias, L.F., Petras, D., Schmid, R., Dührkop, K., Rainer, J., Sarvepalli, A., et al. (2020). Feature-based molecular networking in the GNPS analysis environment. Nat Methods. 17, 905-908.

[The UniProt Consortium](https://pubmed.ncbi.nlm.nih.gov/?term=UniProt%C2%A0Consortium%20T%5BAuthor%5D). (2018). UniProt: the universal protein knowledgebase. [Nucleic Acids Res.](https://www.ncbi.nlm.nih.gov/pmc/articles/PMC5861450/) 46, 2699.

Paradis, E., Claude, J., Strimmer, K., (2004). APE: analyses of phylogenetics and evolution in R language. Bioinformatics. 20, 289-90.

Pluskal, T., Castillo, S., Villar-Briones, A., Oresic, M. (2010). MZmine 2: modular framework for processing, visualizing, and analyzing mass spectrometry-based molecular profile data. BMC Bioinformatics.11,395

Price, M.N., Dehal, P.S., Arkin, A.P. (2015). FastTree 2--approximately maximum-likelihood trees for large alignments. PloS One. 5, e9490.

Zhang, J., Kobert, K., Flouri, T., Stamatakis, A. (2014). PEAR: a fast and accurate Illumina Paired-End reAd mergeR. Bioinformatics. 30, 614-620.

**SUPPLEMENTARY FIGURE AND TABLE LEGENDS**

Supplementary Figure 1. Skin hydration measurement by Corneometer CM 825. The differences of skin hydration between the groups were analyzed via a paired t-test. * p<0.05 compared to baseline. BW: body wash, BL: body lotion.

Supplementary Figure 2. Stacked bar plots show the mean relative abundance of bacterial genera found by 16S rRNA gene sequencing at each time point and treatment group combination (A) and in each individual (B). Within plot legends, prefixes (e.g., g = Genus, p = Phylum) indicate the best classification which could be made confidently while reporting to the genus level.

Supplementary Figure 3. Alpha and beta diversity of skin microbiome of the subjects applied with a prebiotic body wash (BW) and body lotion (BL) or standard body wash and body lotion (control) for 3 and 6 weeks. (A) Alpha diversity assessed by observed operational taxonomic units (OTUs) richness and (B) Shannon diversity index. (C) Differences in beta diversity were visually assessed by Principal Coordinates Analysis based on weighted Unifrac distances.

Supplementary Figure 4. Principal component analysis (PCA) plot using the R package ropls v1.24.0 of metabolic profiles of skin swabs and skincare products characterized by LC-MS/MS Neg (A) and Pos (B). Confidence ellipse level at 95%. BW: body wash, BL: body lotion.

Supplementary Figure 5. Principal component analysis (PCA) plot (A) and Partial least squares-discriminant analysis (PLS-DA) plot using the R package ropls v1.24.0 with 100 random permutations and 5-fold cross-validation (B) of skin metabolic profiles characterized by LC-MS/MS Pos. Confidence ellipse level at 95%. BW: body wash, BL: body lotion.

Supplementary Figure 6. Heatmap illustrating correlation between microbes, clinical outcomes and discriminant metabolite features identified by LC-MS/MS Pos. (A) Spearman’s correlation between discriminant metabolite features and microbes , computed using the rcorr function from the R package Hmisc v.5.0.1 and p-values adjusted using the Benjamini & Hochberg correction.. Correlations having rho > 0.3 (low correlation and above) and q-value < 0.05 are labeled. (B) Pearson’s correlation between discriminant metabolite features and Skin Hydration. Correlations having q-value < 0.05 are labeled. (C) Median peak abundance (normalized, log transformed) per discriminant metabolite feature within each treatment group. Differential abundance significance between Baseline vs. 6 weeks per Control or Prebiotic group is illustrated using * for q-value < 0.05, ** for q-value < 0.01 and *** for q-value < 0.001 (paired Wilcoxon Rank Sum Test, Benjamini & Hochberg adjusted). Cells in the Beauty Product row labeled “P” (Prebiotic) or “C” (Control) suggest the corresponding metabolite at 6 weeks originates from the Beauty Product. BW: body wash, BL: body lotion.

Supplementary Table 1. Bacterial species identified by 16S rRNA gene sequencing and identified by Analysis of composition of microbiota with bias correction (ANCOM-BC) to be discriminant from baseline and after product application in Prebiotic Body wash and body lotion group.

Supplementary Table 2. Bacterial species identified by 16S rRNA gene sequencing and identified by Analysis of composition of microbiota with bias correction (ANCOM-BC) to be discriminant from baseline and after product application in the Control Body wash and body lotion group.

Supplementary Table 3. 96 clinically relevant metabolic features identified by LC-MS/MS Neg. Cohen’s d: the effect size for measuring the difference between baseline and 6 weeks. r_Skin_Hydration: Pearson’s correlation coefficient between skin hydration and metabolite abundance (log10 transform of normalized abundance); p_Skin_Hydration: p-value for the Pearson’s correlation. BW: body wash, BL: body lotion.

Supplementary Table 4. 90 clinically relevant metabolic features identified by LC-MS/MS Pos. Cohen’s d: the effect size for measuring the difference between baseline and 6 weeks. r_Skin_Hydration: Pearson’s correlation coefficient between skin hydration and metabolite abundance (log10 transform of normalized abundance); p_Skin_Hydration: p-value for the Pearson’s correlation. BW: body wash, BL: body lotion.
