# Supplementary figures and images for: Multi-omic approach to decipher the impact of skincare products with pre/postbiotics on skin microbiome and metabolome
Source: Front Med (Lausanne). 2023 Jul 18;10:1165980. doi: 10.3389/fmed.2023.1165980 (PMC10392128; doi:10.3389/fmed.2023.1165980)

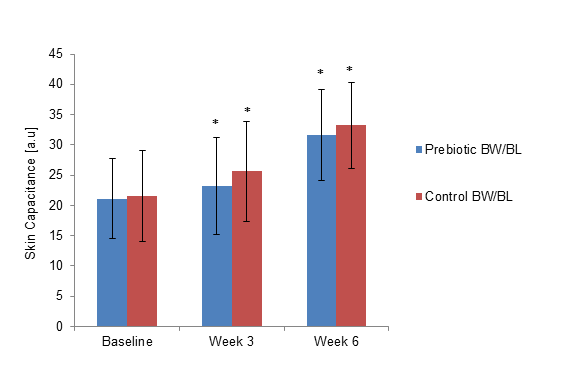

Supplement: Supplementary file 2 [file Image_1.TIF]

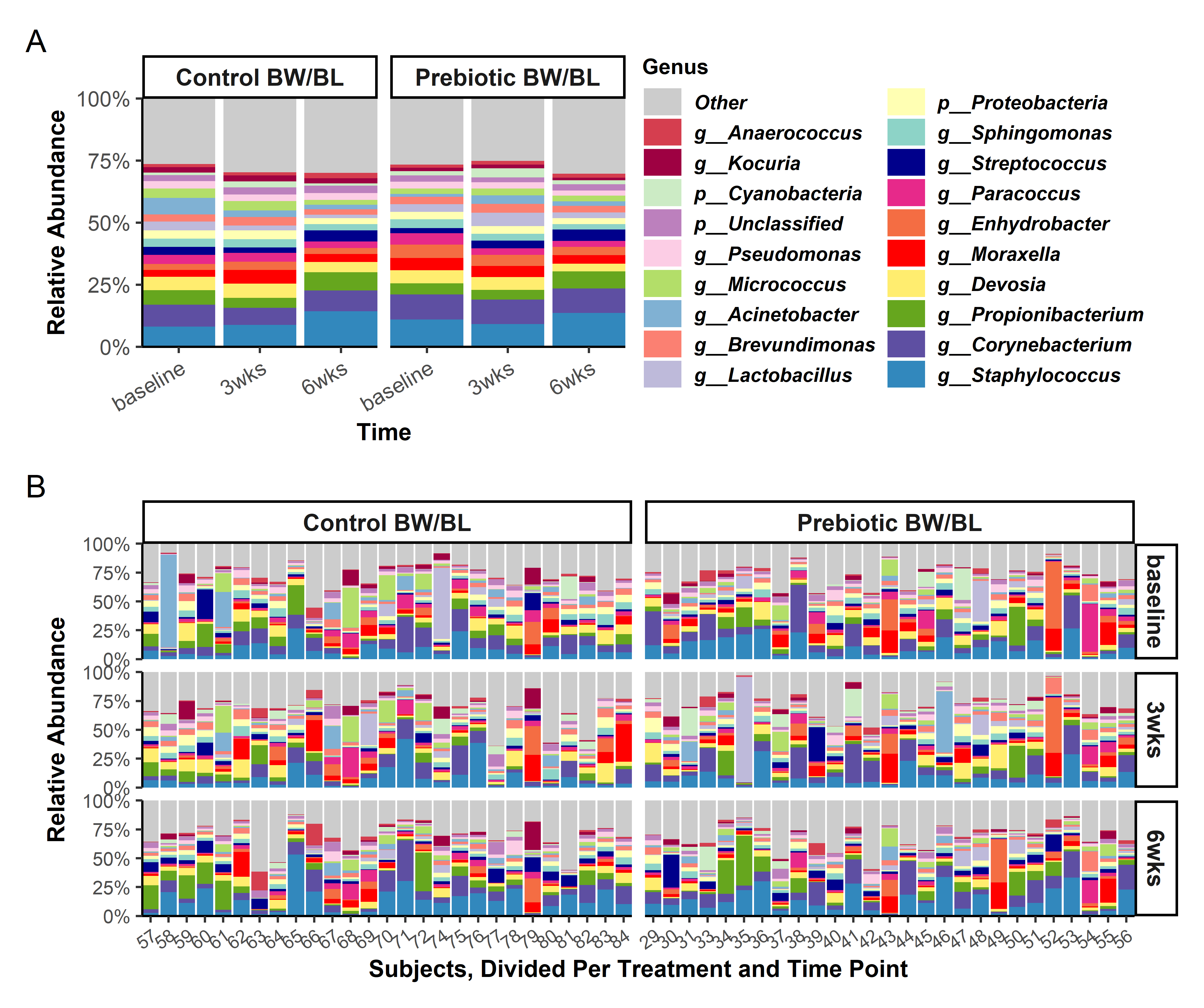

Supplement: Supplementary file 3 [file Image_2.TIFF]

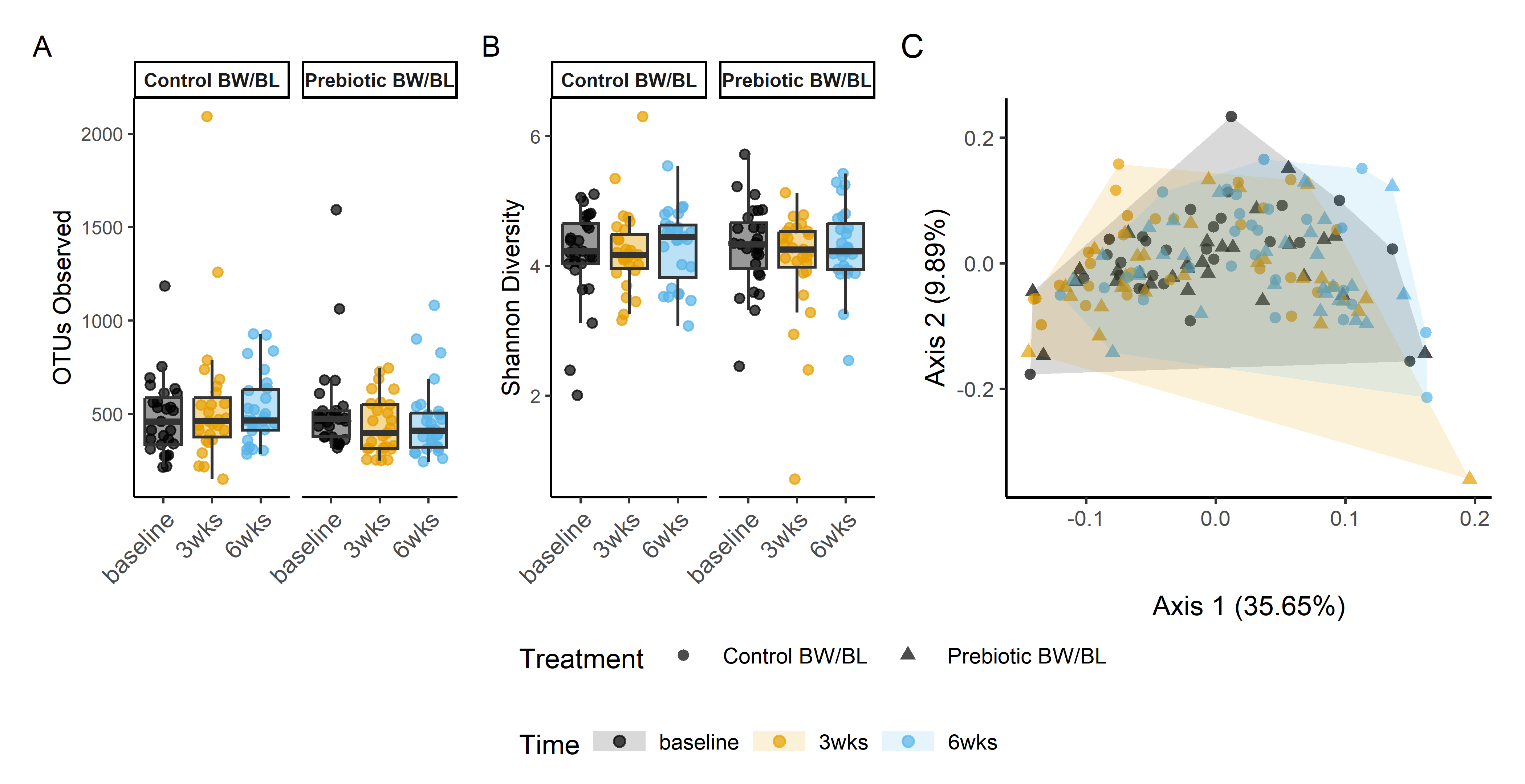

Supplement: Supplementary file 4 [file Image_3.TIFF]

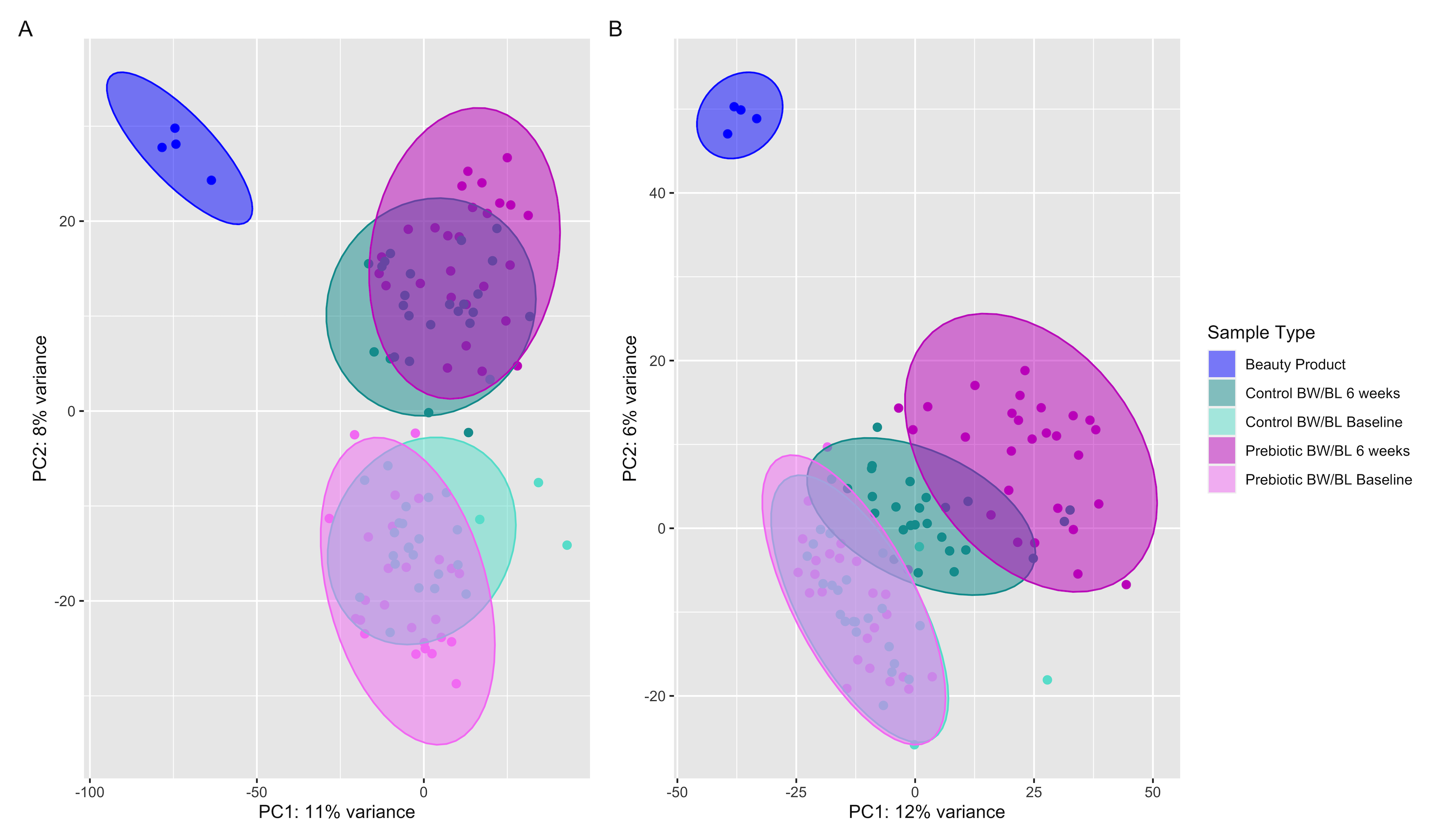

Supplement: Supplementary file 5 [file Image_4.TIFF]

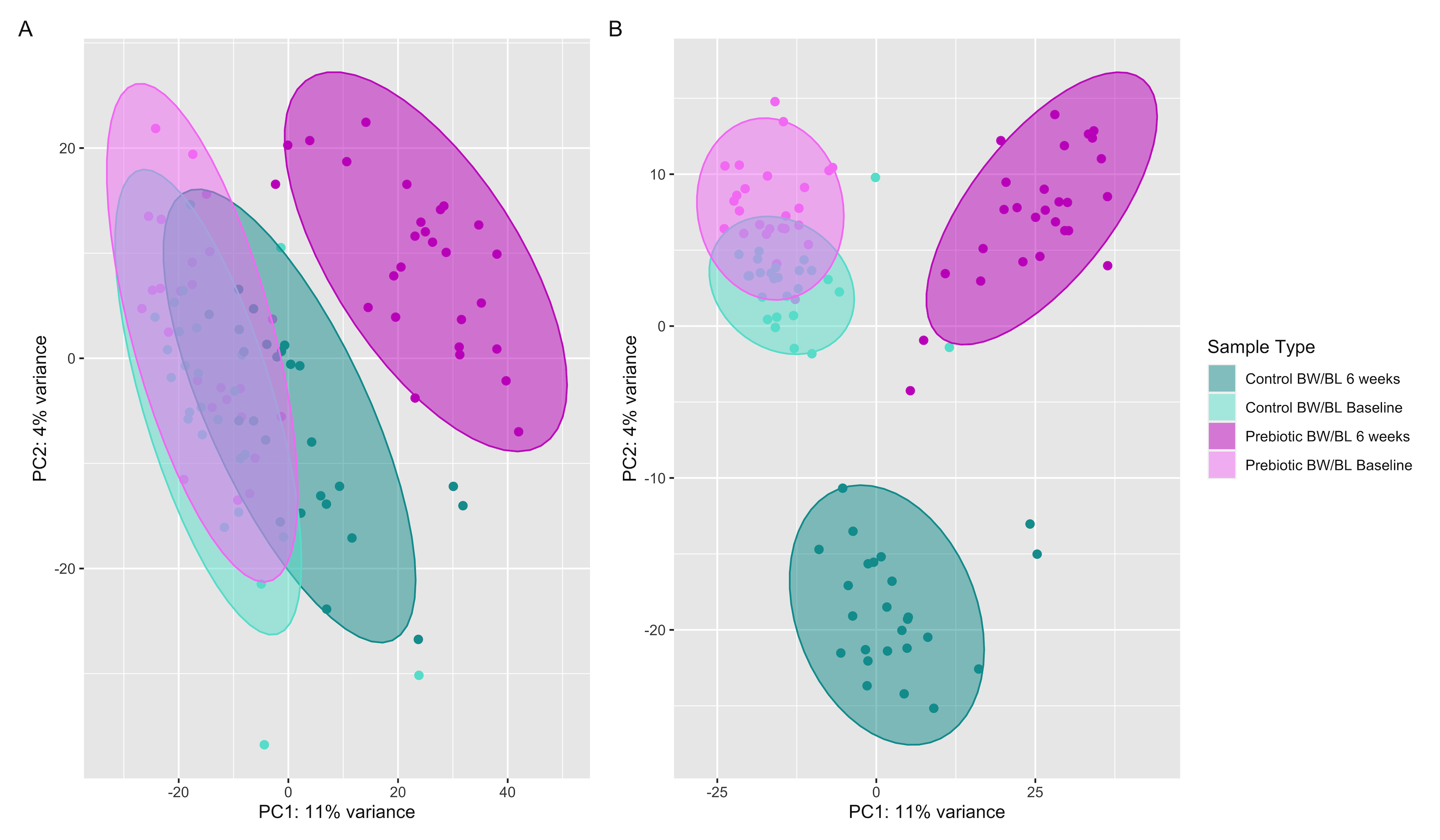

Supplement: Supplementary file 6 [file Image_5.TIFF]

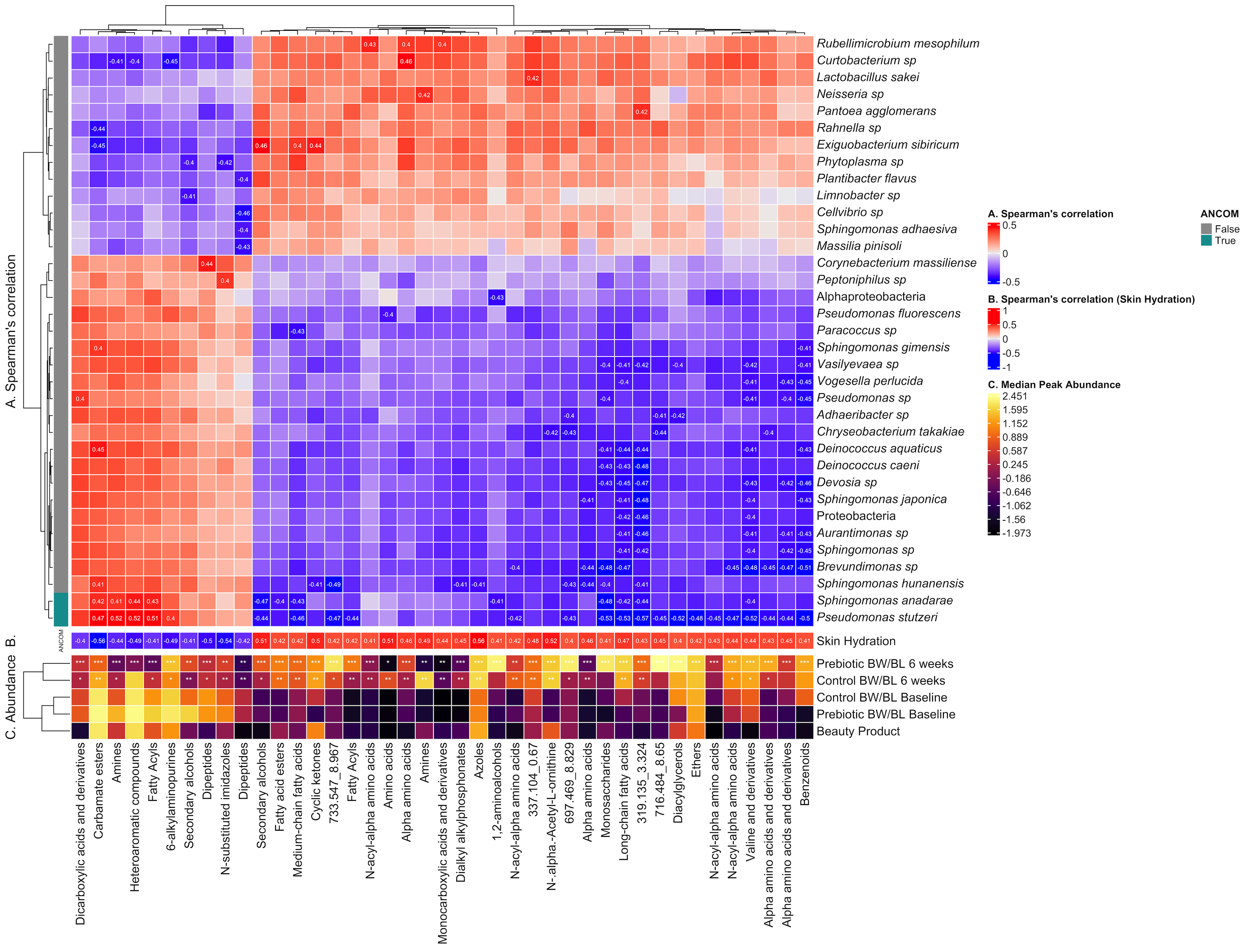

Supplement: Supplementary file 7 [file Image_6.TIFF]
